# A new species of *Nebalia* (Crustacea, Leptostraca) from coral reefs at Pulau Payar, Malaysia

**DOI:** 10.3897/zookeys.605.8562

**Published:** 2016-07-14

**Authors:** B.H.R. Othman, T. Toda, T. Kikuchi

**Affiliations:** 1 Institute of Oceanography and Environment, Universiti Malaysia Terengganu, 21030 Kuala Terengganu, Terengganu, Malaysia; 2 School of Marine and Environmental Sciences, Universiti Malaysia Terengganu, 21030 Kuala Terengganu, Terengganu, Malaysia; 3 Graduate School of Engineering, Soka University, Hachioji, Tokyo 192-8577, Japan; 4 Faculty of Education & Human Sciences, Yokohama National University, 79-2 Tokiwadai, Hodogaya, Yokohama 240-8501, Japan

**Keywords:** Nebalia, new species, Leptostraca, coral reefs, Pulau Payar, Malaysia

## Abstract

A new species of Leptostraca, *Nebalia terazakii* **sp. n.** is described and figured. The species was sampled from the coral reefs of Pulau Payar Marine Park, Langkawi, Malaysia. There are 32 existing species of *Nebalia* but *Nebalia terazakii* sp. n. can be distinguished from the other known species of *Nebalia* by the following combination of characters: the rostrum is 1.89 times as long as wide and the eyes have no dorsal papilla or lobes. Article 4 of the antennular peduncle has one short thick distal spine. The proximal article of the endopod of maxilla 2 is shorter than the distal, a feature peculiar to *Nebalia terazakii* **sp. n.**, the exopod of maxilla 2 is longer than article 1 of the endopod, the posterior dorsal borders of the pleonites 6 to 7 are provided with distally sharp denticles, anal plate with prominent lateral shoulder and finally, the terminal seta of the caudal rami is 1.17 times the length of the entire rami.

## Introduction

The leptostracan genus *Nebalia* was thought to contain only a few species, but with rather a wide range of distribution. However, when Dahl (1985, 1990) re-examined specimens from the European Shelf and the Southern Oceans, he managed to solve some of the long outstanding problems on the taxonomy of *Nebalia*. According to Dahl (1985) the taxonomy of the European species was in a state of confusion and the synonymy so interwoven. Two new species were described for the European Shelf (Dahl 1985), and for the Southern Oceans Dahl (1990) described four new species from specimens previously referred to as *Nebalia longicornis* Thomson, 1879.

Since then many new species of *Nebalia* from various areas namely the Atlantic coasts (Haney et al. 2001; Moreira et al. 2003, 2009), Mediterranean Sea (Ledoyer 1997; Moreira et al. 2007, 2012; Kocak and Moreira 2015), Mexico (Escobar-Briones and Villalobos-Hiriart 1995), Red Sea (Wagele 1983), Africa (Kensley 1976; Olesen 1999, Bochert and Zettler 2012), Pakistan (Kazmi and Tirmizi 1989), New Caledonia (Ledoyer 2000), Hong Kong (Lee and Bamber 2011), South Korea (Song et al. 2012; Song and Min 2016) and California (Martin et al. 1996; Vetter 1996; Haney and Martin 2000, 2005) have been described. The present finding brings the total of the existing *Nebalia* species to 33.

As part of the study on the biodiversity of marine invertebrate fauna around Malaysia (Othman and Morino 1996, 2006; Othman and Toda 2006; Othman and Azman 2007; Gan et al. 2010; Lim et al. 2010, 2015; Azman and Othman 2012, 2013; Chew et al. 2014; Tan et al. 2014) a new species of *Nebalia* from Pulau Payar Marine Park has been discovered. The area has extensive coral reefs and was gazetted a National Park and near the northern entrance of the Straits of Malacca, within the Langkawi group of islands. Pulau Payar is situated 15 km south of the main Langkawi island and 20km off mainland Peninsular Malaysia.

## Materials and methods

The animals were sampled using a baited trap. The trap consists of a clear 500 ml screw-cap wide mouthed polythene jars with a dozen 8 mm holes on the bottle cap. Fresh fish used as bait were wrapped in cheese cloth. Animals caught were fixed in 4% formaldehyde sea water solution and later transferred into glycerol. Drawings were made using a camera lucida on a Zeiss Axioscope light microscope. The specimens were dissected and appendages and mouthparts mounted onto slides in glycerol.

Type materials were deposited in the South China Sea Research and Repository Centre, Institute of Oceanography and Environment, Universiti Malaysia Terengganu, 21030 Kuala Terengganu, Terengganu, Malaysia.

## Results

### Order Leptostraca Claus, 1880 Family Nebaliidae Samouelle, 1819
*Nebalia* Leach, 1814

#### Nebalia terazakii sp. n.

Taxon classificationAnimaliaLeptostracaNebaliidae

http://zoobank.org/E31AE970-6D53-487F-9EC3-472804F76537

**Figure 1.**
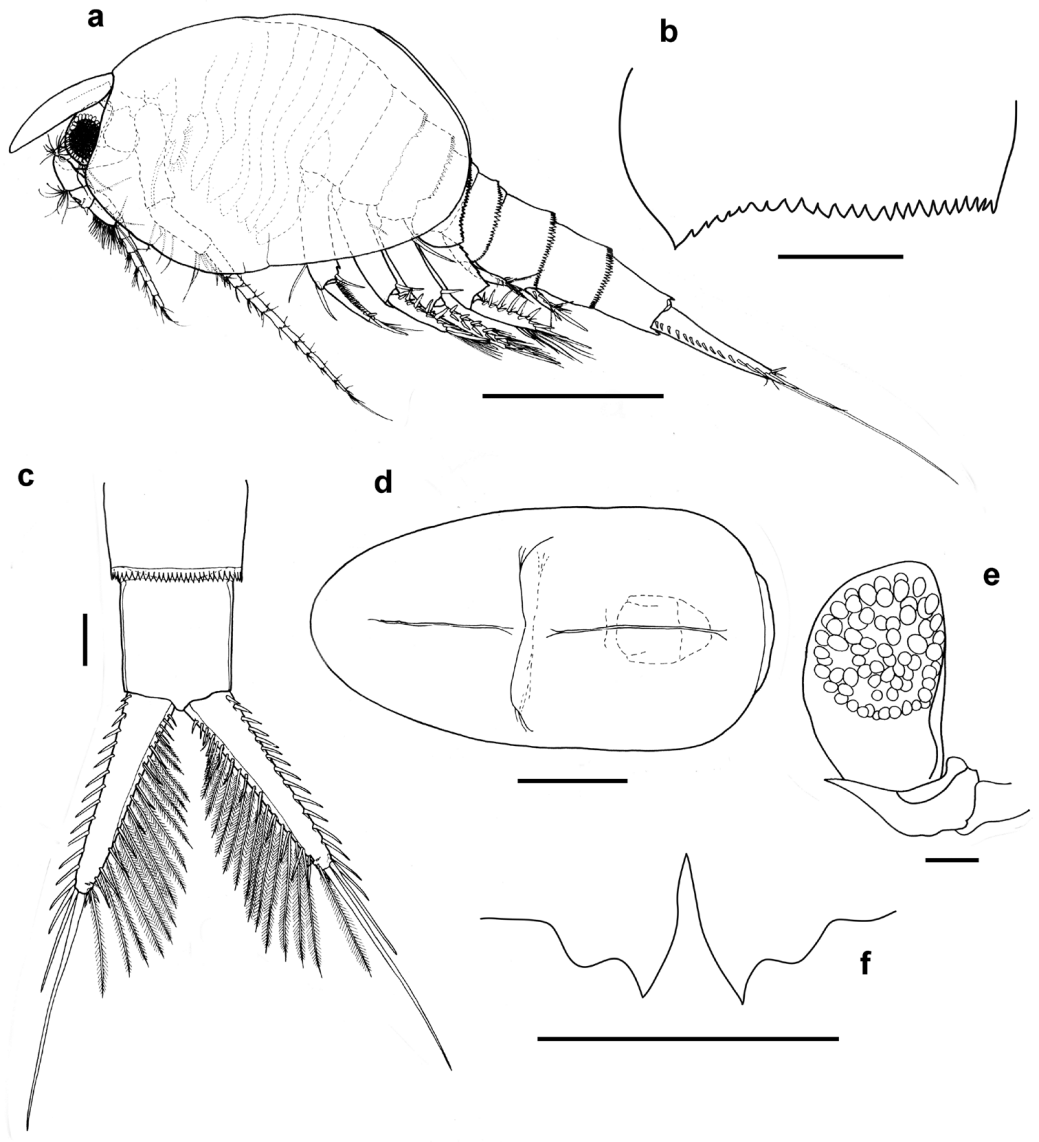
*Nebalia terazakii* sp. n., female, **a** body, lateral **b** epimeron of pleopod 4, lateral **c** caudal furca, dorsal **d** rostrum, dorsal **e** eye, lateral **f** anal scale, ventral. Scale bars: **a** = 1.0 mm, **b–f** = 0.2 mm.

**Figure 2.**
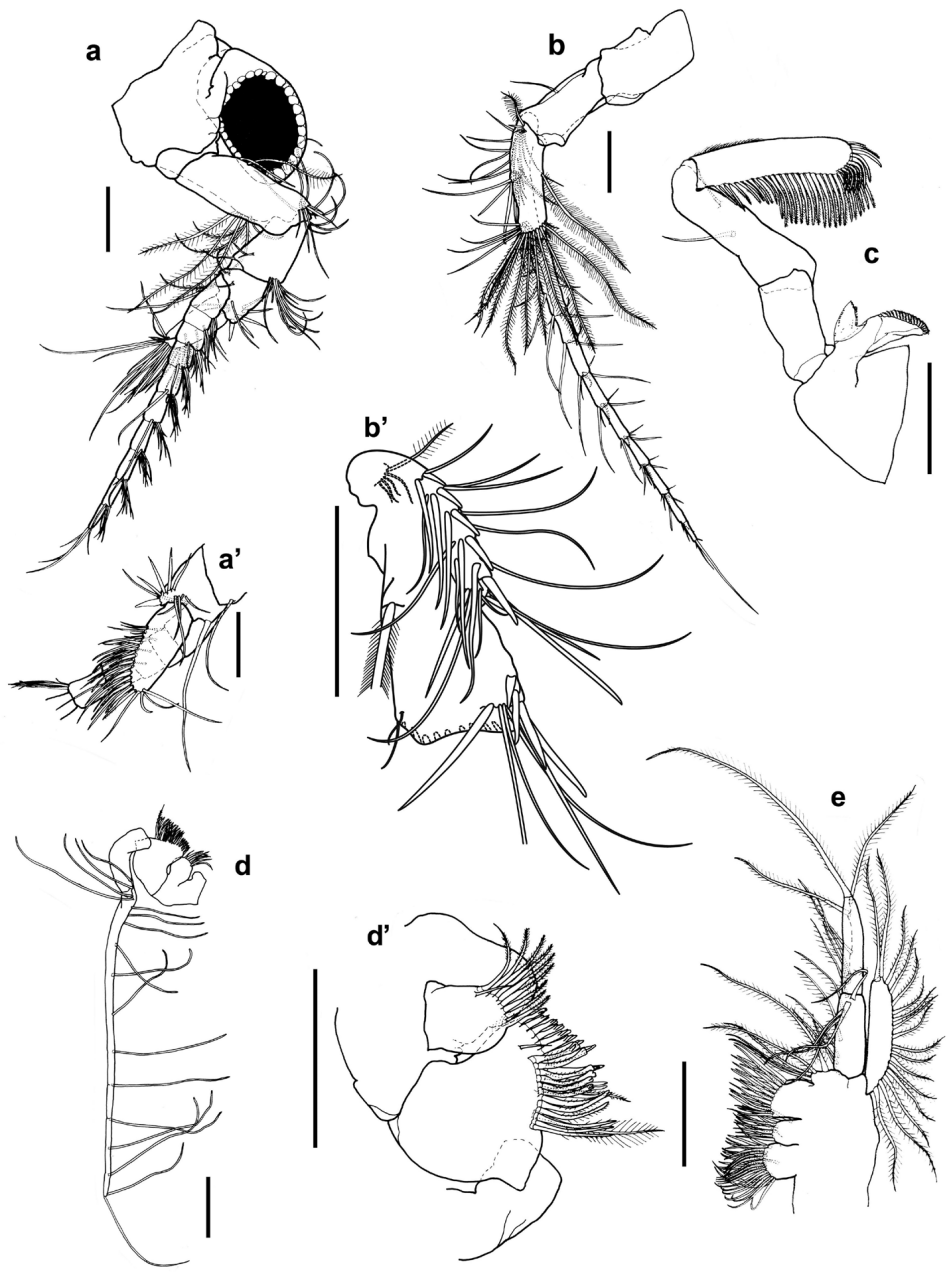
*Nebalia terazakii* sp. n., female, **a** antennule, lateral **a**’ antennule scale **b** antennae, lateral **b**’ antennae, article 3, medial border **c** mandible, anterior: **d** maxilla 1, anterior **d**’ maxilla 1 endite, anterior **e** maxilla 2, anterior. Scale bars: 0.2 mm.

**Figure 3.**
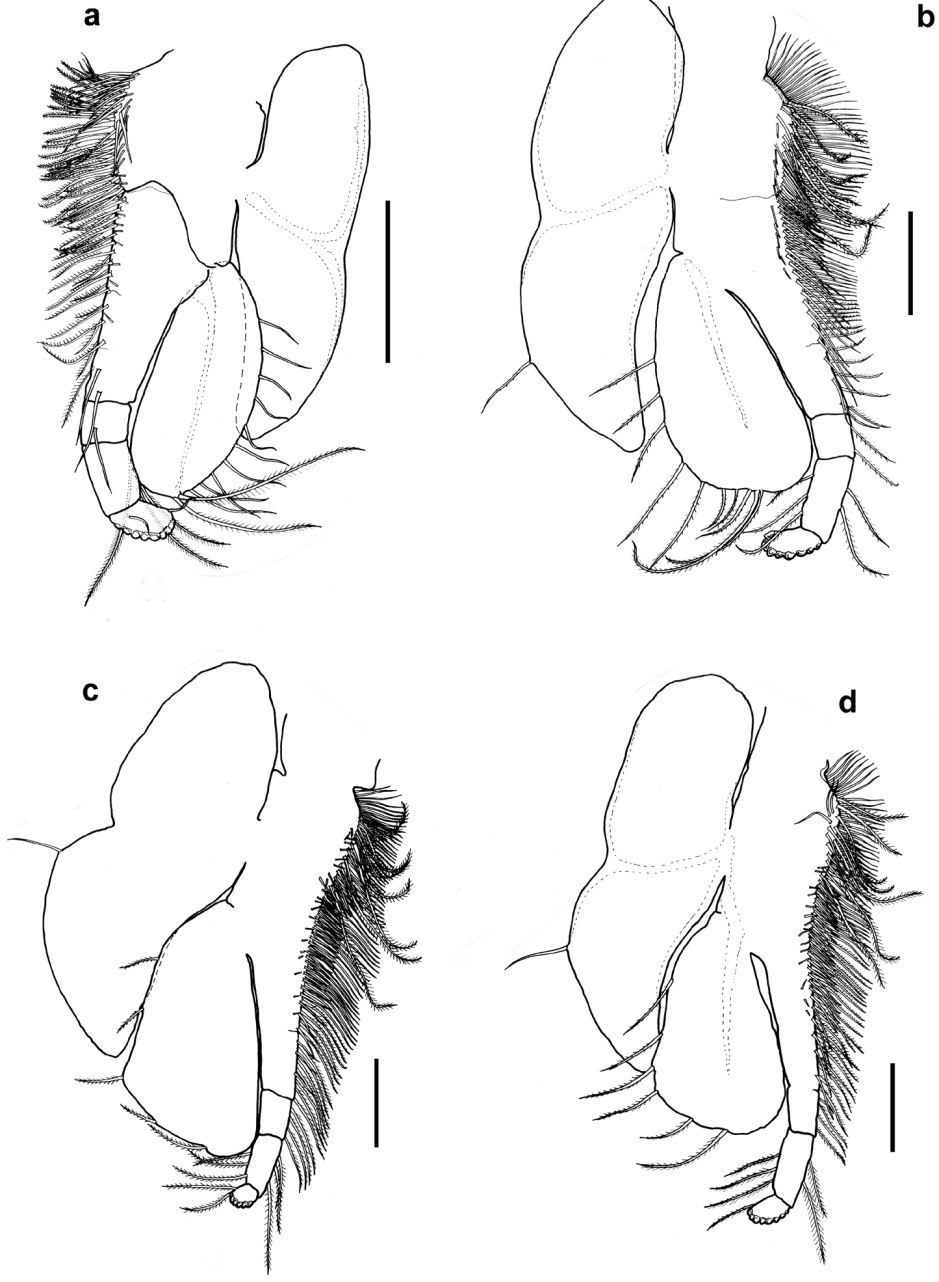
*Nebalia terazakii* sp. n., female, **a** thoracopod 1, dorsal **b** thoracopod 2, dorsal **c** thoracopod 3, dorsal **d** thoracopod 4, dorsal. Scale bars: 0.2mm.

**Figure 4.**
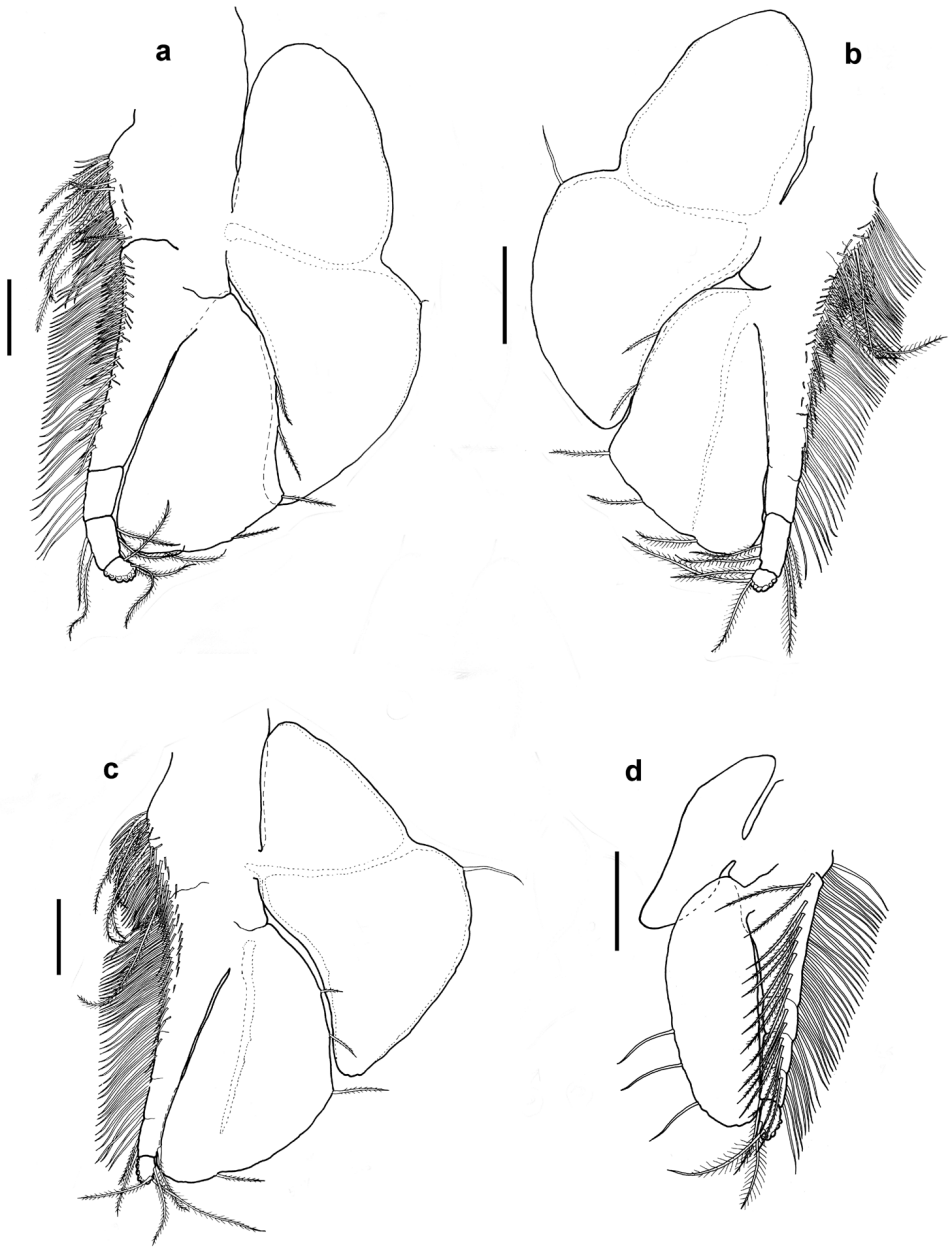
*Nebalia terazakii* sp. n., female, **a** thoracopod 5, dorsal **b** thoracopod 6, dorsal **c** thoracopod 7, dorsal **d** thoracopod 8, dorsal. Scale bars: 0.2 mm.

**Figure 5.**
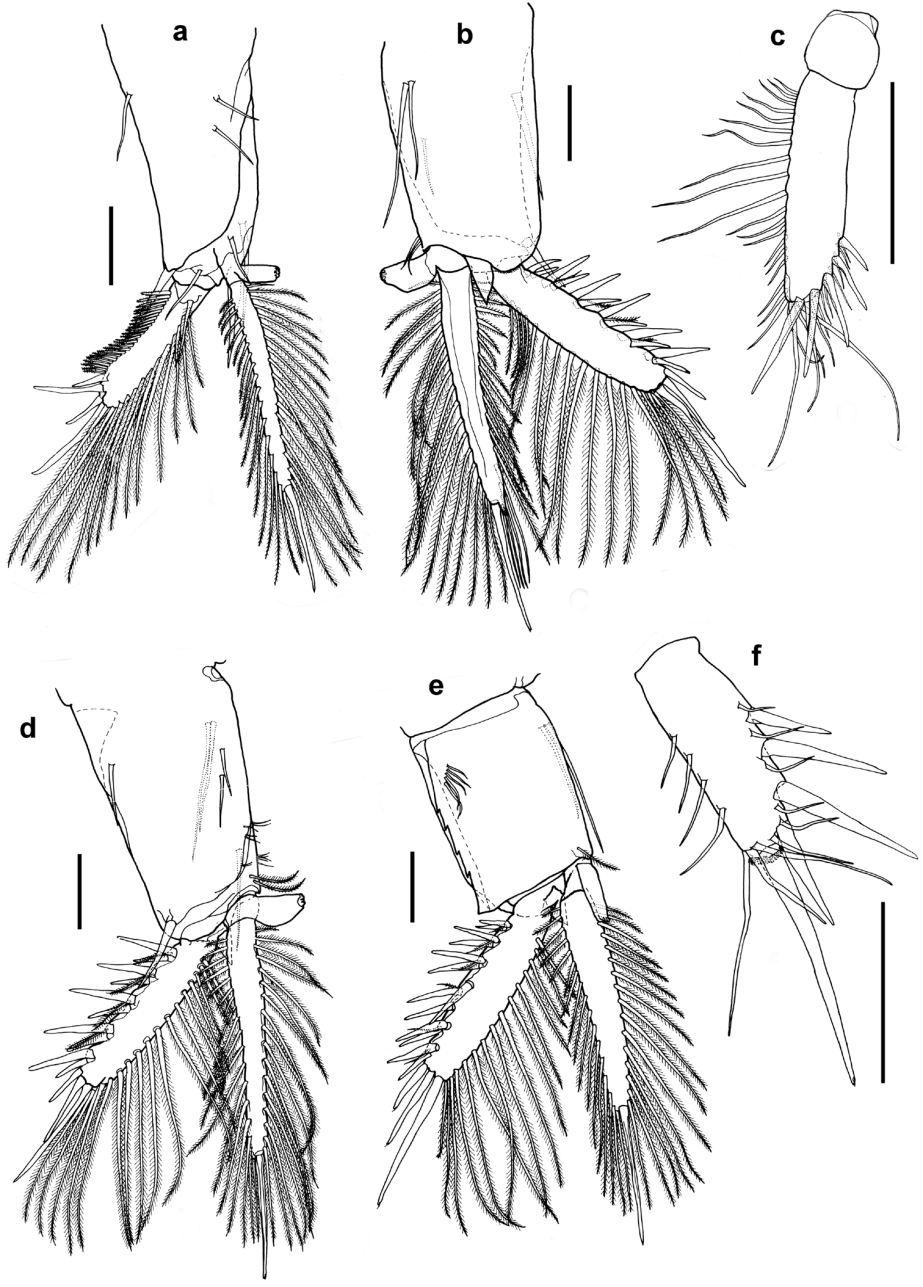
*Nebalia terazakii* sp. n., female, **a** pleopod 1, anterior **b** pleopod 2, posterior **c** pleopod 5, anterior **d** pleopod 3, anterior **e** pleopod 4, anterior **f** pleopod 6, anterior. Scale bars: 0.2 mm.

Figs 1 , 2 , 3 , 4 , 5

##### Material examined.

Holotype: female, post ovigerous, 2.2 mm carapace length and 5.3 mm total length, Ref UMTCrus 00478, sample no 3272; paratypes, 12 adult females, Ref UMTCrus 00479, sample no 3272; 6 adult females, Ref UMTCrus 00480, sample no 3274; 5 adult females, Ref UMTCrus 00481, sample no 3276; 4 adult females and 32 juveniles, Ref UMTCrus 00482, sample no 3277.

##### Type locality.

Pulau Payar, Kedah, Malaysia 6°03'48.0"N, 100°02'28.9"E; baited trap on coral reef, 12.9.1995.

##### Description of holotype.

Body robust (Fig. 1a). Carapace about 1.5 times as long as wide, almost reaching posterior margin of pleonite 4, dorsally convex, anterior and posterior margin rounded.

Rostrum (Fig. 1a, d) prominent, 1.89 times as long as wide, slightly broader near base, sides almost parallel from proximal end to about midway then tapering to rounded distal end, ca. 0.3 times of carapace length, extending beyond eye and anterior margin of carapace; narrow in lateral, upper margin convex, lower margin flattened.

Compound eye without papilla (Fig. 1e), ommatidial part occupying 0.67 length of eyestalk. Eye stalk with small and pointed supraorbital spine present at posterior margin, tip not reaching posterior border of cornea.

Antennule (Fig. 1a) extending to about 0.4 times length of carapace. Peduncle 4-articulate (Fig. 2a), article 1 short, 0.2 times length of article 2, naked. Article 2, three times as long as wide with two plumose setae on mid-anterior margin, row of six plumose setae on lateral margin and 13 setae antero-distally. Article 3 half length of article 2, slightly longer than wide with two setae on disto-lateral margin and an antero-distal cluster of setae. Article 4, 0.7 times length of article 3, width same as length, with one distal stout spine, two rows each of four setae on inner lateral margin near spine. Outer lateral margin with two setae and long distal seta behind the scale. Antennular scale elliptical (Fig. 2a’), 2.5 times as long as wide, with rows of setae on anterior distal margin. Antennular flagellum slightly longer than peduncle and composed of 10 articles.

Antenna (Fig. 1a) extending beyond posterior margin of carapace. Peduncle 3-articulate (Fig. 2b), article 1, 1.59 times as long as wide, naked. Article 2, 0.86 times as long as article 1, 1.94 times as long as wide, and with one seta on anterior margin about midway. Article 3 (Fig. 2b’) longer than article 2, with four short setae and one plumose seta on proximal inner margin and with several rows of setae along medial anterior margin; (1) six simple setae, (2) five short spine along proximal half, (3) six longer simple setae, (4) three thin setae, (5) six long setae; terminal row of four spines, increasing distally in length, the distal most next to four simple setae and one long spine, one long plumose seta on posterior margin about midway, cluster of about 10 plumose setae along distal inner margin, two short setae on postero-distal margin. Flagellum well developed, composed of 11 articles; each article with five terminal setae of various lengths.

Mandible (Fig. 2c) well developed. Mandibular palp three-articulate, article 2 equal in length as article 3, and with sub-terminal seta and another seta midway on lateral margin. Article 3 cylindrical, with marginal setae-row covering anterior margin beginning small distance from proximal margin, all setae equal in length, weakly plumose, beginning with length about as wide as article, doubling in length about 2/5 from proximal end, posterior margin with minute hairs covering about midway from proximal end. Article 1, 0.6 times length of article 3, 1.95 times as long as wide, naked. Molar process three times as long as wide, slightly shorter in length than article 1 of palp. Distal margin with rows of teeth forming grinding surface. Incisor process broad basally with acute terminal process and with minute teeth along inner and outer face.

Maxilla 1 (Fig. 2d) with distal endite as long as proximal one and carrying row of plumose setae and two rows of sculptured setae on inner medial margin (Fig. 2d’). Inner medial margin of proximal endite lobed into two parts, with upper one bearing row of nine weakly plumose setae. Palp very long, about 4.6 times longer than combined length of both endites, and with 16 widely spaced long setae along its length and a terminal seta.

Maxilla 2 (Fig. 2e) protopod with four endites, endites 1 to 3 armed with many rows of short weakly plumose setae, endite 4 with five relatively longer plumose setae. Endopod two-articulate, article 1, 0.83 times length of article 2 and with nine plumose setae on medial margin. Article 2 with six plumose setae on medial margin and one weakly plumose terminal seta 1.67 times combined length of articles 1 and 2. Exopod slightly longer than article 1 of endopod and with one terminal and 16 weakly plumose setae spreading from proximal to distal outer margin.

Thoracopods leaf-like, all eight thoracopods with endopods extending beyond distal margin of exopods, and with terminal article of endopods showing traces of sheded brood pouch setae. Thoracopod 1 (Fig. 3a), exopod elliptical in shape, 2.3 times as long as wide, extending to middle of sub-terminal article of endopod, with 15 weakly plumose setae along outer margin equally spaced from distal to proximal end. Endopod five-articulate with two rows of plumose setae and one row of spines along inner margin from proximal to distal end of article 2, tuft of smooth setae also present near proximal end of endopod. Epipod bilobed and elongated, 3.5 times as long as wide and reaching distal 2/3 of exopod. Thoracopods 2 (Fig. 3b) and 3 (Fig. 3c), exopod triangular in shape with broadest part 1.45 times as long as wide, extending to 0.33 times of sub-terminal article of endopod, and with six to ten plumose setae on outer-lateral margin from 1/3 way of proximal end to its distal end. Endopod four-articulate with row of weakly plumose setae on inner medial margin extending from proximal end of endopod to sub-terminal article, row of shorter setae and row of spines extending from proximal end to about 0.67 times of endopod length. Row of seven plumose setae also present near distal end. Epipod bilobed, slightly broarder than that of thoracopod 1 and 3.2 and 2.8 times as long as wide for thoracopods 2 and 3, respectively. Thoracopod 4 (Fig. 3d) similar to thoracopods 2 and 3 except that endopod three-articulate and exopod extends to proximal end of sub-terminal article of endopod. Thoracopods 5 (Fig. 4a) and 6 (Fig. 4b) similar to preceeding thoracopods except having broarder epipods with 2.3 times as long as wide for thoracopod 5 and 2.1 times as long as wide for thoracopod 6. Endopod of thoracopod 5, four-articulate whereas thoracopod 6, three-articulate. Exopods extend to 0.67 length of sub-terminal article of endopod. Thoracopod 7 (Fig. 4c) similar to preceeding thoracopod except endopod is two-articulate and exopod extend to about 0.67 times of terminal segment of endopod. Distal lobe of epipod much broader, about 1.8 times as long as wide. Thoracopod 8 (Fig. 4d) endopod five-articulate and with row of smooth setae extending from proximal end to distal end of sub-terminal article. A row of plumose setae extends from proximal end of endopod to distal end of sub-terminal article. Exopod oblong 2.7 times as long as wide with three long setae on outer margin. Epipod narrow, 3.4 times as long as wide and extends 0.25 times of exopod.

Posterior margins of pleonites 3 to 7 serrated throughout their lengths, denticles pointed along dorsal margins changing to blunt along lateral margins of pleonites 3 to 6, denticles of pleonite 7 pointed all through. Epimeron of pleon 4 with margin evenly serrated and with acutely pointed posterolateral corner (Fig. 1b).

Pleopod 1 (Fig. 5a), composed of protopod, exopod and endopod. Protopod measuring 1.7 times as long as wide, broadest at proximal end tapering at distal end, with one seta on outer margin 1/3 from proximal end, two setae on inner margin same distance from proximal end and two small setae close to endopod and one stout long distolateral seta reaching to 0.67 times of exopod. Endopod, two-articulate, 0.85 times as long as protopod and 1.5 times longer than exopod, and with long terminal spine half length of endopod, reticulum present. Exopod with comb-row of short trifid setae on outer margin, long plumose setae along inner margin and 4 stout spines on distal margin, terminal spine of which by far largest.

Pleopod 2 (Fig. 5b), protopod 1.7 times as long as wide and with pair of setae on inner lateral margin 1/4 way from proximal end, pair of short setae on distal margin near endopod, stout seta on distal margin near exopod and two setae on outer lateral margin. Endopod two-articulate, subequal in length as protopod with plumose setae along outer and medial margins and terminal spine half as long as endopod, reticulum present. Exopod 0.8 times length of endopod with six pairs of robust setae and single plumose seta in between on outer margin, three terminal setae and row of long plumose setae on medial margin.

Pleopod 3 (Fig. 5d) protopod 1.7 times as long as wide, with pair of setae each on posterior and anterior lateral margins 1/3 way from proximal end, seta on outer margin 1/3 way from proximal end, distal margin with pair of plumose setae near endopod and stout seta near exopod reaching 0.4 times of exopod. Endopod two-articulate, subequal in length as protopod, with plumose setae along outer and medial margins and terminal spine almost half as long as endopod, reticulum present. Exopod 0.7 times length of endopod and with five pairs of stout seta and single plumose seta in between and three terminal stout seta and row of long plumose setae along medial margin.

Pleopod 4 (Fig. 5e) protopod rectangular, 1.3 times as long as wide, outer margin serrated and with row of five setae 1/4 way from the proximal margin, inner margin with pair of setae 1/4 way from proximal end, inner distal margin with single plumose seta. Endopod two-articulate, 1.3 times as long as protopod, with plumose setae along outer and medial margins, terminal spine 0.6 times as long as endopod, rectangular shaped reticulum present. Exopod 0.8 times length of endopod and with seven pairs of stout setae and single plumose seta in between, three terminal stout setae and row of long plumose setae along medial margin.

Pleopod 5 (Fig. 5c) uniramous, two-articulate, distal article 3.7 times as long as wide, with five stout spines along distolateral and terminal border, increasing in length distally, about 25 simple setae along medial and distal border.

Pleopod 6 (Fig. 5f) uniramous, single article, 2.6 times as long as wide, with five very strong lateral and distal stout spines, distal most spine slightly longer than pleopod, with circlet of sharp teeth surrounding base. Lateral border with six setae, medial border with four setae and three distal setae.

Anal somite, anal plate and uropods (Fig. 1c), anal somite (pleonite 8) short, marginally longer than wide, slightly longer than pleonite 7. Anal plates (Fig. 1f) with convex medial margin and with long, acute points over medial part of scale, lateral margin with prominent and narrow shoulder. Uropods, about 0.9 times as long as combined pleonite 7 and anal somite, slightly tapering distally, with about 16 to 18 robust setae along lateral margin progressively increasing in length from proximal to distal end. Along lateral inner margin of uropod, about 12 to 14 similar setae as well as 15 to 18 long plumose setae. Terminal spine of uropod about 1.17 times length of uropod.

##### Remarks.

Dahl (1985) revised the Leptostracans of the European Shelf and described a new genus with *Sarsinebalia typhlops* (G.O. Sars, 1870), formerly *Nebalia typhlops*, and relegated the species *Nebalia geoffroyi* Milne-Edwards, 1928 as a junior synomym of *Nebalia herbstii* Leach, 1814. Martin et al. (1996) expounded *Nebalia pugettensis* (Clark, 1932) as *nomen nudum* and replaced it with *Nebalia hessleri* Martin, Vetter & Cash-Clark, 1996. Walker-Smith and Poore (2001) revised the classification of the Leptostraca using phylogenetic analysis and reducing the number of species in the genus from 41 to 32 known species. Currently, there are 33 described species of *Nebalia* as in Table 1.

**Table 1.** List of existing species of *Nebalia*, type locality and references.

Species	Type locality	Reference
*Nebalia abyssicola* Fage, 1929	Monaco	Moreira et al. (2012)
*Nebalia antarctica* Dahl, 1990	Antarctic, Ross Sea	Dahl (1990)
*Nebalia biarticulata* Ledoyer, 1997	Marseille, France	Ledoyer (1997)
*Nebalia bipes* (Fabricius, 1730)	Greenland	Dahl (1985)
*Nebalia borealis* Dahl, 1985	Norway	Dahl (1985)
*Nebalia brucei* Olesen, 1999	Zanzibar, Tanzania	Olsen (1999)
*Nebalia cannoni* Dahl,1990	South Georgia	Dahl (1990)
*Nebalia capensis* Barnard, 1914	Cape Town, S. Africa	Kensley (1976)
*Nebalia clausi* Dahl, 1985	Adriatic Sea, Italy	Dahl (1985)
*Nebalia dahli* Kazmi & Tirmizi, 1989	Karachi, Pakistan	Kazmi and Tirmizi (1989)
*Nebalia daytoni* Vetter, 1996	San Diego, California	Vetter (1996)
*Nebalia deborahae* Bochert & Zettler, 2012	Namibia & Angola	Bochert and Zettler (2012)
*Nebalia dolsandoensis* Song & Min, 2016	Dolsand Island, S. Korea	Song and Min (2016)
*Nebalia falklandensis* Dahl, 1990	Falkland Is.	Dahl (1990)
*Nebalia gerkenae* Haney & Martin, 2000	Monterey Bay, California	Haney and Martin (2000)
*Nebalia helbstii* Leach, 1814	British coast	Dahl (1985)
*Nebalia hessleri* Martin, Vetter & Cash-Clark, 1996	Southern California	Martin et al. (1996)
*Nebalia ilheoensis* Kensley, 1976	South-western Africa	Kensley (1976)
*Nebalia kensleyi* Haney & Martin, 2005	Marin County, California	Haney and Martin (2005)
*Nebalia kocatasi* Moreira, Kocak & Katagan, 2007	Izmir Bay, Turkey	Moreira et al. (2007)
*Nebalia koreana* Song, Moreira & Min, 2012	Dolsando Island, S. Korea	Song et al. (2012)
*Nebalia lagartensisi* Escobar-Briones & Villalobos-Hiriart, 1995	Yucatan Peninsula, Mexico	Escobar-Briones and Villalobos-Hiriart (1995)
*Nebalia longicornis* Thomson, 1879	South Island, New Zealand	Dahl (1990)
*Nebalia marerubi* Wagle, 1983	Red Sea	Wagle (1983)
*Nebalia mediterranea* Kocak & Moreira, 2015	Aegean Sea Turkey, N. Cyprus	Kocak and Moreira (2015)
*Nebalia melanophthalma* Ledoyer, 2000	Noumea, New Caledonia	Ledoyer (2000)
*Nebalia mortoni* Lee & Bamber, 2011	Hong Kong	Lee and Bamber (2011)
*Nebalia patagonica* Dahl, 1990	Magellan region	Dahl (1990)
*Nebalia pseudotroncosoi* Song, Moreira & Min, 2012	South coast of Korea	Song et al. (2012)
*Nebalia schizophthalma* Haney, Hessler & Martin, 2001	North Atlantic, Gay Head	Haney et al. (2001)
*Nebalia strausi* Risso, 1826	Channel Is, Guernsey	Dahl (1985)
*Nebalia terazakii* sp. n.	Pulau Payar, Malaysia	Present study
*Nebalia troncosoi* Moreira, Cacbelos & Dominguez, 2003	Galicia, Iberian peninsula	Moreira et al. (2003)

*Nebalia terazakii* sp. n. differs from the other known species of *Nebalia* in the following combination of characters: the rostrum is 1.9 times as long as wide, the eyes have no dorsal papilla or lobes, article 4 of the antennule peduncle has only one short thick distal spine, the armature of the external lateral side of the antennal peduncle article 3 has distribution and appearance of spines and setae which differs from other known species, article 1 of the endopod of maxilla 2 is peculiarly short, about 0.83 times as long as article 2, the exopod of maxilla 2 is longer than article 1 of the endopod, the posterior dorsal borders of the pleonites 6 to 7 are provided with distally sharp denticles, anal plate with prominent lateral shoulder and finally, the terminal seta of the caudal rami is 1.17 times the length of the entire ramus. In all other known species of *Nebalia* the proximal article of maxilla 2 is longer than the distal article, however, in *Nebalia terazakii* sp. n. the distal article of maxilla 2 is longer than the proximal, a feature peculiar to *Nebalia terazakii* sp. n.

*Nebalia terazakii* sp. n., when compared with recently described species from the Asian and Southeast Asian regions (Table 2), shows differences in the length to width ratio of the rostrum. The ratios for all species are >2, except *Nebalia melanophthalma* and *Nebalia terazakii* sp. n. which are 1.73 and 1.89 respectively. The area occupied by the ommatidial part of the eye is similar for *Nebalia terazakii* sp. n., *Nebalia dolsandoensis*, *Nebalia melanophthalma* and *Nebalia moretoni*, however, in *Nebalia koreana* and *Nebalia pseudotroncosoi* the area is larger and smaller respectively. Another feature which differs between the species is the number of thick spines on the article 4 antennular peduncle. In *Nebalia melanophthalma* and *Nebalia terazakii* sp. n. there is one thick spine whereas there are >1 for the rest of the species. Article 1 of maxilla 2 endopod is shorter than article 2 in *Nebalia terazakii* sp. n. whereas in all other species articles 1 is longer than 2. Denticles on pleonite 6 to 7 are acutely shaped in *Nebalia melanophthalma* and *Nebalia terazakii* sp. n. but square to rounded in the others. The anal plate shoulder of *Nebalia terazakii* n.sp is prominent and this distinguishes it from the other species mentioned. The uropod and combined pleonite 7 and anal somite length ratios vary between 0.7 and 1.0. The terminal spine to uropod length ratio shows similarity between *Nebalia terazakii* sp. n. and *Nebalia koreana* but differs greatly from *Nebalia dolsandoensis* and *Nebalia moretoni*.

**Table 2.** Comparison of some diognostic characters of *Nebalia* females from the Asian and Southeast Asian regions.

Species	Rostrum length/ width	Area occupied by ommatidial part of eye	Antennule peduncle article 4: no. of thick spines	Maxilla 2 endopod: article 1/ article 2 length	Pleonites 6-7: shape of dorsal denticles	Anal plate shoulder	Uropod length/ pleonite 7 + anal somite	Terminal spine length/ urosome
*Nebalia dolsandoensis*	2.14	0.67	4	1>2	round	present	0.7	1.69
*Nebalia koreana*	2.35	0.85	5	1>2	round	none	0.8	1.15
*Nebalia melanophthalma*	1.73	0.67	1	1>2	acute	none	1.0	na
*Nebalia mortoni*	2.37	0.67	4	1>2	square	none	1.0	1.7
*Nebalia pseudotroncosoi*	2.27	0.5	2	1>2	round to pointed	none	0.9	na
*Nebalia terazakii* sp. n.	1.89	0.67	1	1<2	acute	prominent	0.9	1.17

*Nebalia terazakii* sp. n. is most similar to *Nebalia brucei* in that both species have a broad rostrum with a similar length to width ratio, the antennular armatures on peduncle article 4 are each armed with a single spine, the antennular scales are both elliptical, the epimerons of the pleopod 4 are pointed and the lateral margins of the anal plates are both with prominent shoulder. However, these two species can be distinguished from one another in that the antennular flagellum has 12 articles in *Nebalia brucei*, whereas it is 10 in *Nebalia terazakii* sp. n. The armature of the external lateral side of the antennal peduncle article 3, differ in the distribution and appearance of spines and setae between the two species. Length ratio of maxilla 2 endopod article 1 and 2 is 1.39 in *Nebalia brucei* whereas in *Nebalia terazakii* sp. n. it is 0.83. The terminal spine of uropod is about 1.17 times the length of uropod in *Nebalia terazakii* sp. n. whereas it is 0.70 in *Nebalia brucei*.

##### Etymology.

The species is named after the late Professor Dr. Makoto Terazaki, Ocean Research Institute, University of Tokyo, Japan.

## Supplementary Material

XML Treatment for Nebalia terazakii
